# Caprine MAVS Is a RIG-I Interacting Type I Interferon Inducer Downregulated by Peste des Petits Ruminants Virus Infection

**DOI:** 10.3390/v13030409

**Published:** 2021-03-05

**Authors:** Qiuhong Miao, Ruibing Qi, Chunchun Meng, Jie Zhu, Aoxing Tang, Dandan Dong, Hongyuan Guo, Monique M. van Oers, Gorben P. Pijlman, Guangqing Liu

**Affiliations:** 1 Innovation Team of Small Animal Infectious Disease, Shanghai Veterinary Research Institute, Chinese Academy of Agricultural Science, Shanghai 200241, China; qiuhong.miao@wur.nl (Q.M.); gqyzqrb@gmail.com (R.Q.); mengcc@shvri.ac.cn (C.M.); zj121@shvri.ac.cn (J.Z.); tax1366@163.com (A.T.); dddong2015@126.com (D.D.); guohy9510@163.com (H.G.); 2 Laboratory of Virology, Wageningen University & Research, Droevendaalsesteeg 1, 6708 PB Wageningen, The Netherlands; monique.vanoers@wur.nl

**Keywords:** caprine, mitochondrial antiviral signaling protein (MAVS), Peste des Petits Ruminants Virus (PPRV), innate immunity

## Abstract

The mitochondrial antiviral-signaling protein (MAVS, also known as VISA, IPS-1, or CARDIF) plays an essential role in the type I interferon (IFN) response and in retinoic acid-inducible gene I (RIG-I) mediated antiviral innate immunity in mammals. In this study, the caprine MAVS gene (caMAVS, 1566 bp) was identified and cloned. The caMAVS shares the highest amino acid similarity (98.1%) with the predicted sheep MAVS. Confocal microscopy analysis of partial deletion mutants of caMAVS revealed that the transmembrane and the so-called Non-Characterized domains are indispensable for intracellular localization to mitochondria. Overexpression of caMAVS in caprine endometrial epithelial cells up-regulated the mRNA levels of caprine interferon-stimulated genes. We concluded that caprine MAVS mediates the activation of the type I IFN pathway. We further demonstrated that both the CARD-like domain and the transmembrane domain of caMAVS were essential for the activation of the IFN-β promotor. The interaction between caMAVS and caprine RIG-I and the vital role of the CARD and NC domain in this interaction was demonstrated by co-immunoprecipitation. Upon infection with the Peste des Petits Ruminants Virus (PPRV, genus Morbillivirus), the level of MAVS was greatly reduced. This reduction was prevented by the addition of the proteasome inhibitor MG132. Moreover, we found that viral protein V could interact and colocalize with MAVS. Together, we identified caMAVS as a RIG-I interactive protein involved in the activation of type I IFN pathways in caprine cells and as a target for PPRV immune evasion.

## 1. Introduction

The induction of the interferon (IFN) response is vital for vertebrate hosts to counteract invading viruses and microbial pathogens. In general, cytoplasmic pathogen-associated molecular patterns (PAMPs), such as dsRNA molecules generated during viral infection, are sensed by host pattern recognition receptors, which include toll-like receptors (TLRs), and retinoic-acid inducible gene I (RIG-I)-like receptors (RLRs) and nucleotide-binding oligomerization domain-like receptors (NLRs) [1]. Once invading pathogens are recognized, these receptors activate downstream signal transduction pathways.

RLRs constitute a critical group of intracellular viral RNA sensors that share similar structures and functions: RIG-I, melanoma differentiation-associated gene 5 (MDA5), and Laboratory of Genetics and Physiology 2 (LGP2). RLRs contain an ATP-dependent RNA helicase domain that is required for double-stranded RNA binding and ATP hydrolysis [2]. RLRs share the ability to detect distinct viral RNA structures as it has been shown for many RNA viruses, including retrovirus, influenza virus and Sendai virus, and for some DNA viruses, such as Epstein–Barr virus, and adenovirus [3]. RIG-I and MDA5 each possess two N-terminal tandem caspase activation and recruitment domains (CARDs) that mediate the signaling to Mitochondrial Antiviral Signaling protein (MAVS), leading to the activation of IFN-mediated antiviral immunity. LGP2, on the other hand, lacks CARDs and is thought to have a regulatory function [4].

MAVS functions as the central signaling protein in the RLR-mediated innate immunity pathway and signals to downstream cytokine production, including type I IFN and ultimately IFN-stimulated genes (ISGs). MAVS is also referred to in the literature as IPS-1, VISA and CARDIF [5,6,7,8], and orthologs have been found in several vertebrate species such as mouse [9], tree shrew [10], cats [11], and goose [12], but also in mollusc [13]. The deletion of MAVS has been shown to significantly reduce the levels of antiviral and other pro-inflammatory cytokines normally induced by virus infection [14]. The central role of MAVs in host antiviral immunity is further illustrated by the fact that double-knockout mice (MAVS−/−) are highly susceptible to multiple RNA virus infections [15].

Structurally, MAVS contains an N-terminal CARD-like region, a Proline-Rich region (PRR), and a C-terminal transmembrane (TM) domain, of which the CARD-like and TM domains have been demonstrated to be essential for MAVS signalling in human cells [5]. Initially, MAVS was reported to predominantly localize in mitochondria, where it maintains the stability and function of these organelles [5]. In addition, MAVS was detected in peroxisomes. Upon viral infection, peroxisomal MAVS provides short-term protection by rapidly inducing IFN-independent expression factors, whereas mitochondrial MAVS has been associated with the activation of a more stable antiviral response involving IFN production [16]. During viral infection, the cellular localization of MAVS may change. It has been shown that, following infection with several RNA viruses, mitochondrial-associated endoplasmic reticulum membrane (MAM) was recruited by RIG-I, which then bound to MAVS, and in that way, coordinated the cellular localization of MAVS to regulate the activation of an effective antiviral response [17]. Functional differences among mitochondria MAVS, peroxisomal MAVS, and MAM localized MAVS suggest that the MAVS protein could recruit distinct cellular components, depending on its cellular localisation and as a consequence may form a powerful network during the innate immune response [18].

Viruses have been shown to apply different strategies to counteract MAVS-mediated signaling, and as a consequence, evade the associated innate immune responses. An example is the hepatitis C virus (HCV) NS3/4A protease, which localizes between the MAM and mitochondria, and particularly targets MAVS that is associated with MAM. Since MAM-localized MAVS has been recognized as being capable of transducing RIG-I signalling, HCV NS3/4A protease is predicted to interrupt RIG-I mediated signalling by cleaving MAVS [17].

Thus, MAVS is a central protein in the induction of type I IFNs in a number of mammals, but data for goats are missing thus far. Infections with the morbillivirus, Peste des Petits Ruminant Virus (PPRV, family *Paramyxoviridae,* genus *Morbillivirus*) are characterized by conjunctivitis, rhinitis, pneumonia and stomatitis, and enteritis, and reach mortalities as high as 90% in small ruminants. PPRV replication occurs in the respiratory tract, where antigen-presenting cells (APC) (or dendritic cells (DC), macrophages and monocytes) are infected and lymphocytes play a major role during immune responses [19]. PPRV causes immunosuppression in its natural hosts. The virus counteracts the IFN response and signalling pathways. Even though IFNs and ISGs are important during innate immune response, PPRV controls the induction of type I IFNs [20]. To better understand the pathogenesis of PPRV infection and other caprine-related infectious diseases we aimed to characterize the function of caprine (ca) MAVS as a central protein during the innate immune response in caprine cells and analyse its role in type I IFN induction. Furthermore, we investigated whether PPRV infection affected caMAVS levels and whether specific viral proteins interacted with caMAVS. We found that the viral protein V could interact and colocalize with MAVS. By expanding our insight in the mode of action of PPRV in the suppression of innate immunity, we aim to contribute to the control of viral diseases of goats, in particular in PPRV and related morbillivirus infections.

## 2. Materials and Methods

### 2.1. Cloning and Sequence Analysis of Caprine MAVS

Total RNA from immortalized caprine endometrial epithelial cells (EECs) was extracted by using TRIzol reagent (Invitrogen, Carlsbad, CA, USA). Total RNA concentration, as well as the 260/280 and 260/230 ratio, were measured by spectrophotometer (Biotek, Winooski, VT USA). Then cDNA was generated by using M-MLV reverse transcriptase (Promega, USA) with random primers (Invitrogen). Primers (Supplementary Materials Table S1) were designed for amplification of caMAVS and caRIG-I based on the predicted sequence in GenBank (accessions XM_018057391.1 and XM_005683566.3). PCR amplification was performed in a reaction mixture of 50 μL containing TransStart^®^ FastPfu Fly DNA polymerase (Transgen Biotech, Beijing, China). The PCR products were purified on spin columns from Sangon Biotech (Shanghai, China) and cloned to pEASY^®^-BLUNT Zero Vector (Transgene Biotech, China). Inserts of five positive clones were sequenced (Sangon Biotech, Shanghai, China). To analyze the evolution of caprine MAVS, a phylogenetic tree was constructed based on the DNA coding sequence of MAVS proteins from 19 animal species selected as listed in Table S2. The phylogenetic tree was built with the neighbor-joining method with 1000 bootstrap replications by MEGA5.0. The amino acid sequence of caMAVS and those of MAVS from other species, such as human, rabbit, mouse, and sheep, were aligned using the MEGA 5.0 program and edited by the MegAlign package of Lasergene.

### 2.2. Construction of Expression Plasmids

Expression plasmids of Myc-tagged caMAVS and caRIG-I were constructed by inserting the full-length open reading frames (ORFs) into the pCMV-MYC vector with MYC fused to the N-terminus (Clontech, Takara Bio, Kusatsu, Shiga, Japan). HA and 3*Flag-tagged caMAVS constructs were cloned with Infusion technology (Vazyme Biotech Co., Ltd., China) into pCMV-HA (N-Terminal) (Clontech, Japan) and p3*FLag-CMV-10 (Sigma-Aldrich, USA) vectors, respectively. Truncated forms of caMAVS, which lacked the CARD domain (residues 10–77), PRR domain (residues 78–199), Transmembrane (TM) domain (residues 495–517), or Non-Characterized (NC) domain (residues 201–494) were constructed by using Infusion technology or overlapping extension PCR, as described previously [21]. Flag-tagged human MAVS (huMAVS) plasmid was used as a control and was kindly provided by Dr. Yuzhi Fu from the Wuhan Institute of Virology, Chinese Academy of Sciences. The PRDIII/I-luc and PRDII-Luc plasmids were purchased from Beyotime. pRL-TK (Promega Company, USA) expressing Renilla luciferase was used as endogenous transfection control to allow normalization. 

### 2.3. Cell Culture, Transfection, and Virus Infection

Caprine EECs were kindly provided by Prof. Yaping Jin from the Northwest Agricultural University of China and were grown in Dulbecco’s Modified Eagle Medium/F-12 (Thermo Fisher, Waltham, MA, USA) with 10% fetal bovine serum (Gibco, Thermo Fisher). HEK-293T cells were grown in Dulbecco’s Modified Eagle Medium with 10% fetal bovine serum (Gibco, USA). Vero-SLAM cells expressing SLAM receptors were generated at the Shanghai Veterinary Research Institute and grown in Dulbecco’s Modified Eagle Medium with 10% fetal bovine serum (Gibco, USA). Vero-SLAM cells support efficient replication of PPRV and the downstream signaling from caMAVS is functional. In a previous study, we demonstrated that nucleolin could inhibit PPRV replication in Vero-SLAMs [22]. All cells were grown with 1% antibiotics of penicillin and streptomycin (P/S) at 37 °C and with a 5% atmospheric CO_2_ concentration. For transfection experiments, cells were seeded in 6-well plates (NEST Biotechnology, Jiangsu, China) overnight, and transfected with indicated expression plasmids and 36 h post transfection, cells were harvested and used for further experiments.

The PPRV vaccine strain, Nigeria/75/1 (GenBank: HQ197753), was obtained from the Shanghai Veterinary Research Institute cell culture collection and amplified on Vero-SLAM cells. After experiments were completed, all experimental materials were autoclaved at 120 °C for 30 min to kill PPRV. The laboratory was confirmed to P2 (BSL-2) laboratory requirements and all experiments were completed under P2 laboratory conditions.

### 2.4. Quantitative Real-Time PCR (qRT-PCR)

qRT-PCR was performed to measure the mRNA levels of selected ISGs, including caprine RSAD2 (caRSAD2), caprine OASL (caOASL2), caprine IFITM3 (caIFITM3), caprine MX1 (caMX1). The caprine GAPDH was used as endogenous control and for normalization of the data. Gene-specific primers were designed by using Primer3 software or according to previous reports [23] and then synthesized by Sangon Biotech, China. Total RNA was extracted by using the TRIzol Reagent (Invitrogen), and cDNA synthesis was performed using M-MLV reverse transcriptase (Promega, USA) with Oligo dT primer or Random primers (Invitrogen). qRT-PCR was performed using SYBR Premix Ex Taq reagents (Takara, Dalian, China) and StepOne Real-Time PCR Detection System (Thermo Fisher, USA). Fold expression was presented to show the relative abundance of the mRNAs by using the comparative CT (ΔΔCT) method. All experiments were performed in triplicate and all experiments were carried out with at least three repeats.

### 2.5. Reporter Plasmids and Luciferase Assays

HEK-293T cells or EEC Cells (1.25 × 10^5^) were cultured in 24-well plates. Transfections were performed with different amounts of expression plasmid for the full-length caMAVS to compare the activity of stimulating IFN-β-luc, and the same amount of full-length or mutant caMAVs to compare their difference in stimulating IFN-β-luc. Each caMAVS plasmid was co-transfected with the same amount of luciferase reporter plasmid and the pRL-TK plasmid, which is used for data normalization. Transfection with different amounts of expression plasmid for the full length caMAVS (250 ng, 500 ng, 1 μg) was used to compare the induction of IFN-β-luc. The same amount of DNA was used to compare full-length caMAVS with the deletion mutants. The same amount of pRL-TK plasmid (40 ng/well) for data normalization was co-transfected along with 500 ng of firefly luciferase reporter constructs (IFN-β-luc or PRD-III/I-luc and PRD-II-luc). Cells were collected at 24 h post-transfection. Luciferase activity was measured by using the Dual-Luciferase Reporter Assay kit (Promega, USA) according to the manufacturer’s protocols. All reporter assays were repeated at least three times. The luciferase activity of each sample was normalized to the Renilla luciferase activity and all data were expressed as mean relative luciferase with standard deviation (SD). Furthermore, all experiments were performed at least three times independently.

### 2.6. Confocal Immunofluorescence Microscopy

To investigate the subcellular localization of the caprine MAVS protein, Vero-SLAM cells were plated on 15 mm cover glasses (NEST Biotechnology, China) and were transfected with Flag-tagged caMAVS (full-length or mutant versions) or together with the mitochondrial indicator plasmid pDsRed2-Mito (Addgene plasmid # 55838) at 60~70% confluence. Vero-SLAMs were selected for transfection instead of EECs because of the much higher transfection efficiency to achieve sufficient levels of simultaneous expression of two plasmids in one cell. The cells were harvested at 24 h post-transfection and fixed with 4% paraformaldehyde in PBS, and subsequently blocked with 5% Bovine Serum Albumin (BSA) in PBS at 37 °C for one hour. Immunofluorescence analysis was performed with primary antibodies (1:200, see next paragraph) directed against the tags and Alexa Fluor-488-conjugated goat anti-mouse antibody (Invitrogen, USA) or Alexa Fluor-633-conjugated goat anti-rabbit antibody (Invitrogen, USA) (1:1000) as secondary antibodies, respectively. The cell nucleus was stained with DAPI (Beyotime, China) for 5 min and then washed four times with PBS-Tween. The images were taken with a Zeiss LSM880 confocal microscope and analysed by Zen Blue software (Zeiss, Germany). 

### 2.7. Immunofluorescence and Western Blot Analysis

Anti-PPRV-N monoclonal antibody directed against the nucleocapsid protein (N) of PPRV was generated by GenScript (Nanjing, China). Flag-tag primary antibodies were purchased from Sigma (USA). Myc-tag primary antibodies were purchased from Santa Cruz (USA), HA-tag primary antibodies were purchased from Abcam (UK). GAPDH and β-actin antibody were purchased from CoWin Biosciences (China). At 24–36 h after transfection with expression plasmids (caMAVS, huMAVS, or truncated caMAVS), cells were washed thoroughly with cooled PBS and lysed in cell lysis buffer containing a protease inhibitor cocktail (Merck Millipore, Darmstadt, Germany). The lysates were subjected to SDS-PAGE and transferred to nitrocellulose membranes (Whatman, Maidstone, UK). The membranes were then blocked at 37 °C with 5% skimmed milk for an hour and incubated with specific primary antibodies (1:1000) overnight at 4 °C and then subjected to horseradish peroxidase (HRP)-conjugated secondary antibodies (Jackson Immuno Research Laboratories) (1:10,000) for 1 h at room temperature. The bound secondary antibodies were visualized with enhanced chemiluminescence (ECL) (Thermo Fisher Scientific, Pittsburgh, PA, USA). For the virus infection experiment, cell lysates were harvested with indicated time intervals after infection and generated by adding 5×loading buffer to the collected cells and separated by SDS-PAGE and subjected to Western blot analysis. By using indicated primary antibodies which recognized different cellular proteins downstream of the PPRV-induced RIG-I pathway, including MAVS (Cell Signaling Technology, Danvers, MA, USA), TBK1 (CST, USA), P-TBK1 (CST, USA), the different protein expression levels were analyzed in the presence or absence of a PPRV infection. Fis-1 (CST, USA), which is a component of a mitochondrial complex, was used as indication for mitochondria.

### 2.8. Co-Immunoprecipitation

Co-immunoprecipitation (Co-IP) was performed to confirm the interaction between caRIG-I and caMAVS, and to determine whether PPRV protein V (PPRV-V) interacts with caMAVS. HEK-293T cells were seeded on 100 mm dishes and transfected with 5 μg of the Myc-tagged caRIG-I encoding plasmid together with 5 μg of HA-tagged wild-type (WT) or truncated caMAVS (caMAVS-ΔCARD, caMAVS-ΔPRR, caMAVS-ΔNC or caMAVS-ΔTM) encoding plasmids. Alternatively, Flag-V was co-transfected together with Myc-caMAVS. Then, 24 h post-transfection, cells were lysed with cell lysis buffer (150 mM NaCl, 50 mM Tris-HCl, pH 8.0, 5 mM EDTA, 0.5% NP-40) containing protease inhibitors (Merck-Millipore). Lysates were centrifuged at 12,000× *g* for 10 min to remove the cell debris. Precipitation was performed overnight at 4 °C with anti-Myc mAb conjugated to Resin Agarose beads (Thermo Fisher Scientific). The beads were washed four times with lysis buffer and the bound proteins were eluted with SDS loading buffer by heating for 10 min. Fifty microliter volumes of cell lysate were eluted and then subjected to Western blot analysis using specific antibodies.

### 2.9. Statistical Analysis

All statistical analysis was performed using GraphPad Prism Version 5. Data were expressed as means ± standard deviations. A one-way ANOVA test was used to compare multiple groups (>2). *p* values < 0.05 were considered to indicate significant differences.

## 3. Results

### 3.1. Cloning and Sequence Analysis of Caprine MAVS Gene

**Figure 1:**
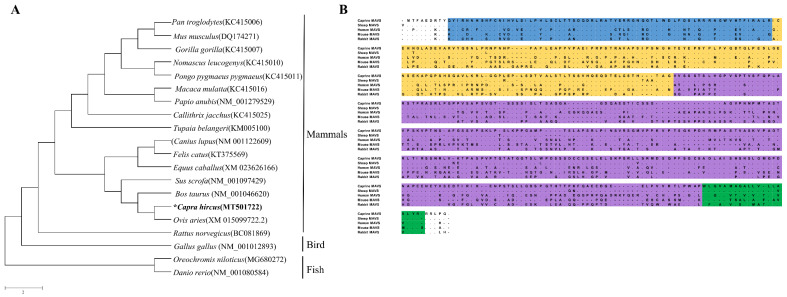
Identification of caprine MAVS. (**A**) Multiple sequences of MAVS from different species were selected to construct the phylogenetic tree. The tree was constructed by using the neighbour-joining method by MEGA5.0 and the scale bar is 2. The amino acid sequences of MAVS used in this program were listed in Table S2. The asterisk indicates caprince MAVS (**B**) Amino acid alignments of caprine, sheep, human, mouse and rabbit MAVS. Sequence alignments were performed and edited with the Lasergene MegAlign program. Structural caspase activation and recruitment domain (CARD), proline-rich region (PRR), non-characterized (NC) and C-terminal transmembrane (TM) domains are indicated in blue, yellow, purple, and green, respectively.

A 1566 bp-long caMAVS cDNA sequence was amplified by conventional RT-PCR. The nucleotide sequence of *Capra hircus* MAVS (caMAVS) has been submitted to GenBank (accession number: MT501722). The predicted 522 amino acid caMAVS protein shares the highest (98.1%) amino acid similarity with the predicted sheep MAVS (XM_015099722.2). A neighbor-joining phylogenetic tree was constructed based on selected cDNA sequences of MAVS from several species (Figure 1A). The phylogenetic tree contains two major branches of mammalian MAVS, which are separated from avian and fish MAVS. In the MAVS amino acid sequence alignment, it was shown that caMAVS has the same structural domains as MAVS found in other mammalian species (Figure 1B).

### 3.2. Schematic Representation and Identification of Caprine MAVS and Its Mutants

**Figure 2:**
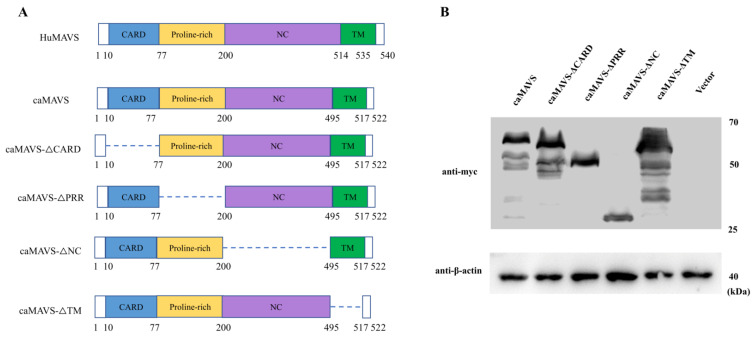
Schematic diagram of caprine MAVS mutants and their expression. (**A**) Schematic representation of caprine MAVS was presented based on the conserved points according to human MAVS. The CARD, PRR, NC and TM domains are represented in blue, yellow, purple, and green, respectively. The schematic representation is also used for constructing truncated mutants. The indicated numbers represent amino acid positions. (**B**) Western blot analysis of the expression of caMAVS and its mutants by transfection with caMAVS and mutant plasmids in HEK-293T cells.

Online structural analysis software (https://www.expasy.org/ (accessed on 1 December 2020)) predicts that caMAVS is a membrane-bound protein with a single C-terminal transmembrane (TM) domain. Based on the comparison with human MAVS and the predicted domains therein, in-frame deletion mutants of caprine MAVS were constructed to analyze the role of the CARD-domain, as well as the PRR, NC, and TM domains (Figure 2A). Western blotting was used to confirm the expression of caMAVS and its mutants before Luciferase and immunofluorescence assay (IFA) were conducted (Figure 2B).

### 3.3. Subcellular Localization of Caprine MAVS

**Figure 3:**
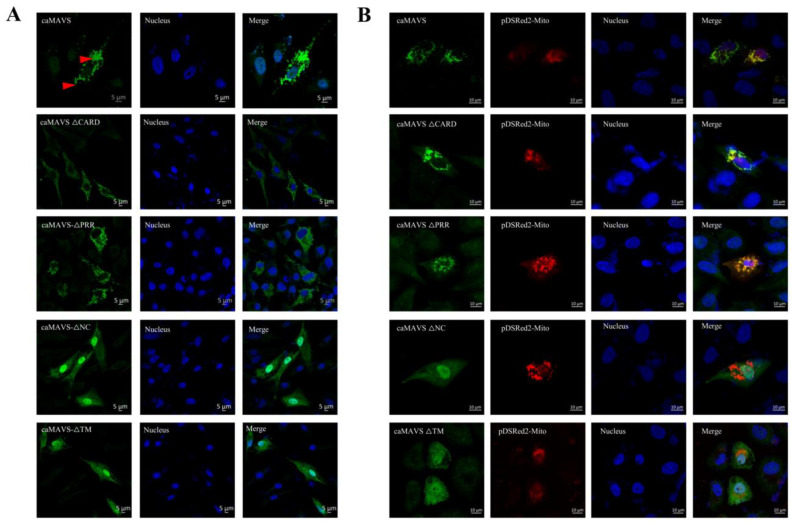
Localization study of caMAVS and its mutants. (**A**) Expression plasmids encoding caMAVS and its different deletion mutants were transfected into Vero-SLAM cells. At 36 h post transfection, cells were fixed and stained with Myc primary antibodies and secondary Alexa-488 labeled antibodies. (**B**) To verify the colocalization of caMAVS (green) and Mitochondria (red), expression plasmids encoding Myc tagged caMAVS and its mutants were transfected together with pDsRed2-Mit. At 36 hpt, cells were fixed and stained as in panel A.

Vero-SLAM cells were transfected with expression plasmids encoding caMAVS and mutants and subjected to IFA to determine the effect of the deletions on the subcellular location of caMAVS. As shown in Figure 3A, full-length caMAVS was abundantly present in the cytoplasm and had a punctate localization. The mutants caMAVS-△CARD and cAMAVS-△PPR also had a punctate localization like the full-length caMAVS. However, when the TM or the NC domains were deleted, the mutant proteins lost their punctate localization and were dispersed over both the cytoplasm and the nucleus. 

As the distribution of caMAVS to mitochondria is vital for its role in signaling, the subcellular location of caMAVS and the various mutants was compared with that of a co-transfected pDsRed2-Mito marker designed for fluorescently labeling mitochondria. As shown in Figure 3B, full-length MAVS co-localized with the mitochondrial marker, as well as caMAVS-△CARD and caMAVS-△PRR. For caMAVS-△TM the mitochondrial localization was disrupted, suggesting that the mitochondrial colocalization was dependent on the TM domain. The caMAVS-△NC mutant, from which a large (295 aa) central part of caMAVS was deleted, did also not colocalize with the mitochondrial marker.

### 3.4. Overexpression of caMAVS-Induced IFN-β via the NF-κB and IRF-3-Mediated Pathways

**Figure 4:**
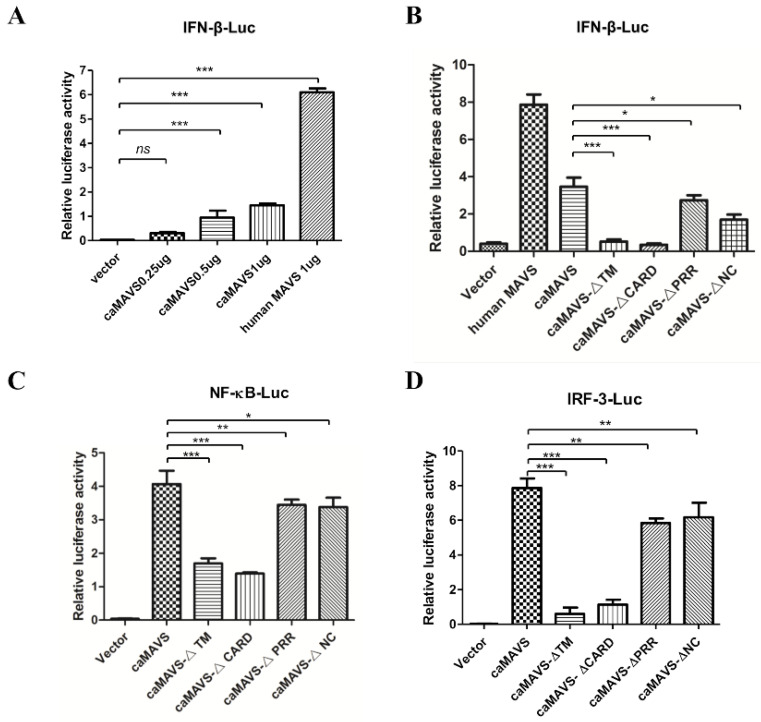
Overexpression of caMAVS induced IFN-β via the NF-κB and IRF-3-mediated pathways. (**A**) HEK-293T cells were transfected with increasing amount of caMAVS, IFN-β-Luc together with endogenous control pRL-TK Plasmid (40 ng/well). (**B**–**D**) HEK-293T cells were transfected with caMAVS or its mutants (500 ng) along with PRDII-Luc (NK-κB-luc), PRDI/III-luc (IRF3-luc)) or IFNβ-Luc together endogenous control pRL-TK Plasmid (40 ng/well). Human MAVS was used as a positive control. At 36 hpt, the HEK-293T cells were lysed, and Rluc and Fluc activities were evaluated using the Promega Dual-Luciferase Reporter Assay System. Furthermore, all the experiments were performed at least three times to ensure the results consistency (The data represent the mean ± SD of three independent experiments. One-way ANOVA was used for statistical analysis; * *p* < 0.05; ** *p* < 0.01; *** *p* < 0.001).

As one of the most important adaptor molecules, MAVS is hypothesized to be critical for virus-induced type I IFN expression in goats, as a link between RLRs and downstream NF-κB and IRF3 activation [5]. To confirm that caMAVS is functional in the type I IFN pathway, HEK293T cells were co-transfected with plasmids expressing caMAVS and human IFN-β promoter-driven luciferase. All cells were also co-transfected with pRL-TK encoding Renilla luciferase to allow for data normalization. As expected, overexpression of caMAVS activated the IFN-β promoter in a dose-dependent manner (Figure 4A). However, the observed expression of luciferase was lower than with human MAVS, which acted as a positive control in this study. 

The next experiment illustrated that caMAVS mutants lacking either the CARD or the TM domains were unable to activate the IFN-β promotor (Figure 4B), in line with previous analysis of mutants of feline MAVS [11]. In the next experiment, we used a set of luciferase report plasmids containing IRF-3 and NF-ĸB-binding motifs (PRDIII/I-Luc, referred to as IRF-3-Luc and PRDII-Luc, referred to as NF-κB-Luc) to further increase our understanding about caMAVS as an adaptor protein. HEK 293T cells were transfected with caMAVS, or mutant versions thereof, together with one of the luciferase reporter plasmids. The results demonstrated that overexpression of caMAVS, as well as the mutants caMAVS-△PRR and caMAVS-△NC, could activate NF-κB and IRF-3 pathways, while caMAVS-△TM and caMAVS-△CARD had lost this ability (Figure 4C,D). Together, the data demonstrated that caMAVS could activate the IFN-β promoter through both NF-κB and IRF-3 signaling. 

### 3.5. caMAVS Overexpression Upregulates the mRNA Level of Caprine ISGs

**Figure 5:**
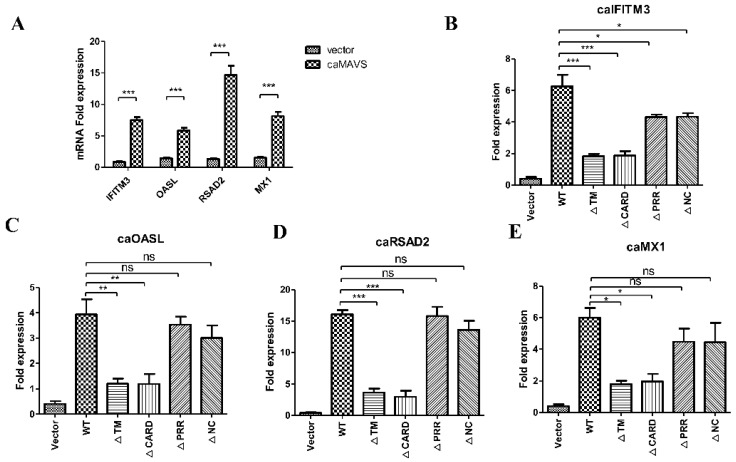
Evaluation of ISGs by overexpression of caMAVS and its mutants in EECs. (**A**) EECs were transfected with either empty vector or caMAVS. The relative mRNA levels of selected caprine ISGs (IFITM3, OASL, RASD2, MX1) were analyzed by qRT-PCR. (**B**–**E**) EECs were transfected with either empty vector or caMAVS and its mutants. The relative mRNA levels of selected ISGs were analyzed by qRT-PCR. Data presented were from at least three independent experiments. Significance was analyzed by GraphPad prism 5.0 software with a one-way ANOVA test (One-way ANOVA was used for statistical analysis; * *p* < 0.05; ** *p* < 0.01; *** *p* < 0.001).

IFN production results in the expression of IFN-stimulated genes (ISGs), which have an important role in morbillivirus antiviral innate immunity [23]. To further establish the role of caMAVS in ISGs induction, the effect of overexpression of caMAVS or its mutants on the transcription of a number of ISGs (caIFITM3, caOASL, caRSAD2, caMX1) was explored by qRT-PCR. Transfection of caMAVS in EECs resulted in the upregulation of the mRNA expression levels of all tested ISGs (Figure 5A). Furthermore, the deletion of either CARD or TM domains significantly limited the induction level of these ISGs (Figure 5B–E). All findings demonstrated that the CARD and TM domains are both essential for the MAVS-medicated IFN production.

### 3.6. caMAVS Interacts with caRIG-I through Its CARD and NC Domain

**Figure 6:**
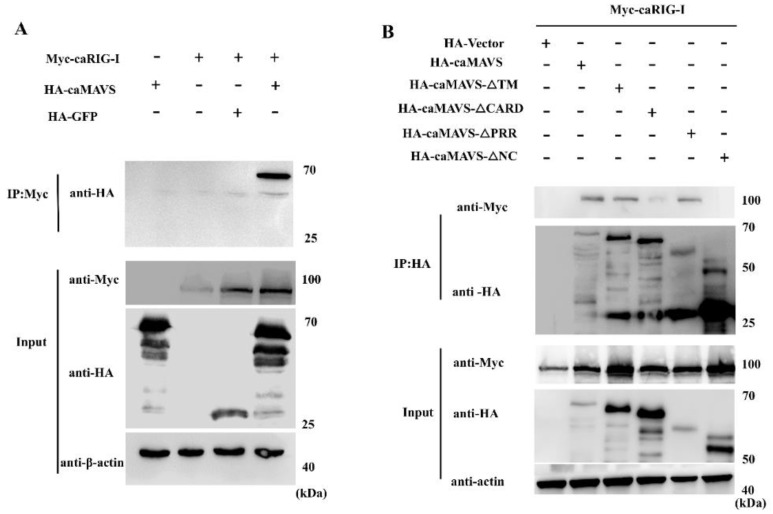
Identification of the interaction between caMAVS and caRIG-I by Co-IP. (**A**) HEK-293T cells seeded in 10 cm dishes and were transfected with HA-tagged MAVS or HA-GFP together with (without) Myc-RIG-I when cells were at around 60% confluency, respectively. Cells were harvested after 24 h post transfection. Cell lysates were precipitated with anti-Myc mAb resin overnight at 4 °C and precipitated proteins detected with anti-HA and anti-Myc mAbs. β-actin was used as protein loading control. (**B**) HEK-293T cells were seeded in 10 cm dishes and were transfected with HA-tagged MAVS and its mutants together with Myc-RIG-I when cells were around 60% confluency. Cells were harvested after 24 hpt and cell lysates were precipitated with anti-HA mAb resin overnight at 4 °C and precipitated proteins were detected with anti-HA and anti-Myc mAb. β-actin was used as protein loading control.

To verify that caMAVS functions as the adaptor of RIG-I, the caprine RIG-I gene was amplified and subcloned into pCMV-Myc (Clontech, USA) and tested in co-immunoprecipitation assays. HEK293T cells were transfected with HA-caMAVS (or its mutants), together with Myc-caRIG-I, and cell lysates were analyzed at 36 h post transfection. Myc-caRIG-I was precipitated with anti-Myc conjugated resin beads and Myc-caRIG-I and HA-caMAVS were detected using anti-bodies against the Myc and HA-tags, respectively. As shown in Figure 6A, the protein samples were incubated with Myc antibody and then the elution was subjected for HA antibody check. The results confirmed the interaction between caRIG-I and full-length caMAVS. Moreover, as shown in Figure 6B, the mutants with PPR or TM domains deleted still interacted with caRIG-I. On the other hand, the CARD domain deletion mutant displays a strongly reduced interaction with caRIG-I, indicating that the CARD domain of MAVS plays a key role in this interaction. Interestingly, also the NC domain mutant, which did not colocalize with mitochondria in the fluorescence studies also lost the ability to interact with caRIG-I. 

### 3.7. caMAVS Response to PPRV Infection in EEC Cells 

**Figure 7:**
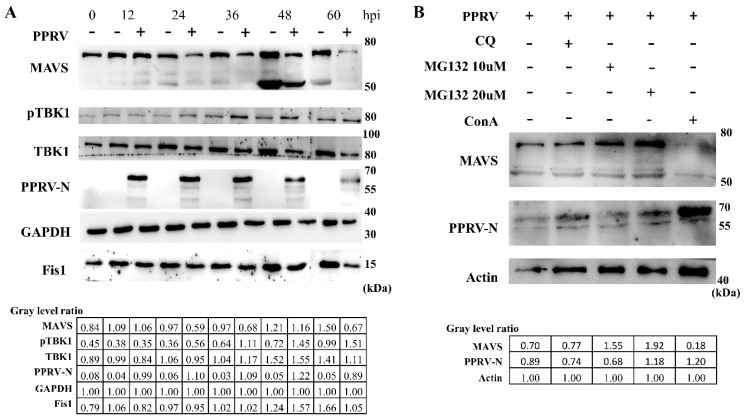
caMAVS is degraded through the proteasome pathway during PPRV infection. (**A**) EECs were infected with the PPRV vaccine strain, Nigeria/75/1, at MOI 10. The relative protein level of several important molecules during viral infection was checked by WB. (**B**) Several inhibitors were used after infection of PPRV on EECs to demonstrate which pathway is involved in MAVS degradation. The relative level of individual proteins was quantified with respect to β-actin.

PPRV has been responsible for severe infectious disease in small ruminants, including goats and sheep. The virus caused significant economic losses in the goat and sheep industry and also threatens wildlife conservation [24,25]. To determine where caMAVS functions as a key factor in the antiviral innate immune response to PPRV, EEC cells were infected with PPRV. Through screening for several proteins known to be important for the induction of innate immunity, we found that caMAVS was degraded after virus infection, as shown in Figure 7A. The endogenous protein expression level of the 70 kDa MAVS was significantly decreased following infection with a high dose of PPRV. The 70 kDa MAVS was downregulated, while the expression level of a smaller (45–50 kDa) band reactive with caMAVS antibodies was upregulated at 48 h with/without virus infection for reasons unknown. A truncated MAVS isoform was also identified in other species but was unable to trigger IFN production [26]. Furthermore, we observed that caMAVS mRNA levels remained relatively stable after PPRV infection (data not shown), which is consistent with other studies. In infections with other viruses, MAVS cleavage was a result of apoptosis-activated degradation [27], autophagic degradation [28] or ubiquitin-proteasome pathways [29]. Several inhibitors have been widely used to elucidate which pathway is involved in MAVS cleavage. MG132 is a commonly used proteasome inhibitor, chloroquine (CQ) is a classic inhibitor of autophagy, and Concanavalin A (ConA) induces autophagy. In our study, we found that the proteasome inhibitor MG132 was able to rescue caMAVS expression to mock-infection levels, in contrast to the lysosome inhibitor chloroquine (CQ). ConA treatment markedly reduced MAVS expression levels. Overall, this suggests that caMAVS degradation during PPRV infection is dependent on the ubiquitin-proteasome pathway (Figure 7B).

### 3.8. PPRV V Protein Interacted with caMAVS

**Figure 8:**
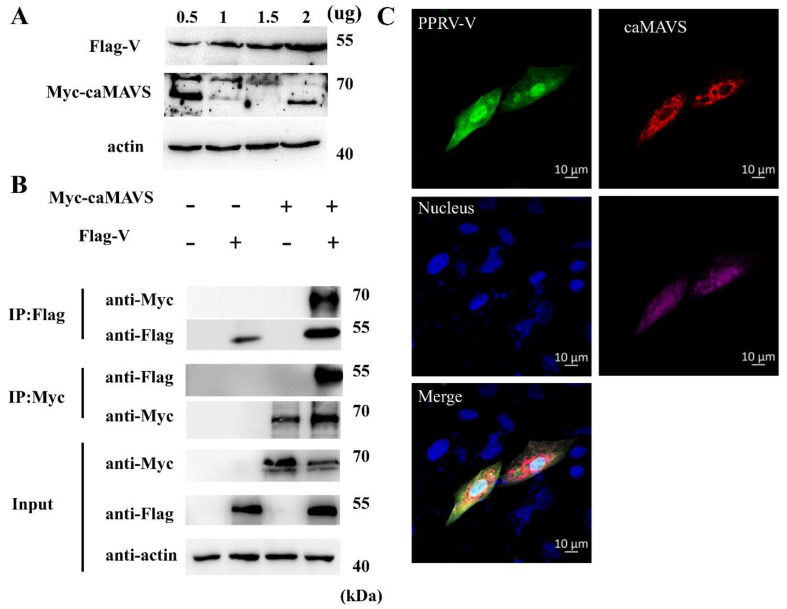
caMAVS interacts and colocalizes with PPRV viral protein V. (**A**) Increasing amounts of plasmids encoding Flag-tagged PPEV protein V (Flag-V) (0.5 ug, 1 ug, 1.5 ug, 2 ug) were co-transfected with plasmid-encoding Myc-tagged caMAVS (2 ug) in 6-well plates. PPRV V and caMAVS were detected by Western blot using β-actin as loading control. (**B**) Co-IP was performed to identify interaction between PPRV V and caMAVS. Flag-V and Myc-caMAVS plasmids were transfected into HEK-293T cells independently or together. Cell lysates were subjected to immunoprecipitation with anti-Myc or anti-Flag mAb overnight at 4 °C. The expression of the transfected proteins was determined by Western blotting by anti-Flag and anti-Myc mAbs, respectively. (**C**) The colocalization study was performed by transfection of Myc-caMAVS (red), Flag-V (green) together with pDsRed2-Mito (pink) and subjected for IFA with anti-Flag and anti-Myc mAbs as primary antibodies.

To identify a putative viral protein responsible for caMAVS degradation in PPRV-infected cells, HEK-293T cells were co-transfected with viral protein-encoding plasmids V and Myc-caMAVS because Newcastle Disease Virus (NDV) V could target MAVS degradation to inhibit host type I interferon production [30]. Clearly, caMAVS expression level was greatly reduced during co-transfection with PPRV-V (Figure 8A). Co-IP assays between with the flagged tagged V protein of PPRV and Myc-caMAVS demonstrated that PPRV-V and caMAVs co-immunoprecipitated, irrespective of which of the two proteins was targeted for pull-down, indicative of a (direct) interaction between these two proteins (Figure 8B). Furthermore, Flag-V co-localized with Myc-caMAVS and with the mitochondrial marker pDsRed2-Mito (Figure 8C) when overexpressed in Vero-SLAM cells, indicating that PPRV-V most likely acts on mitochondrial MAVS. 

## 4. Discussion

PPRV is the causative agent of a highly contagious disease that affects both domestic and wild small ruminants [24]. Except for farm animals, studies also reported the infection of PPRV lineage II in the Chinese water deer, *Hydropotes inermis* [31] and recent outbreaks of PPRV have also been reported to occur in other wild animals in different countries [32,33]. PPRV appears to induce immune suppression during the acute phase of the disease [34], which may favor the establishment of secondary infections with other pathogens. Recently, it was demonstrated that PPRV utilizes the viral Nucleoprotein (N) and Phosphoprotein (P) to inhibit interferon signalling by blocking the JAK-STAT Pathway [35]. Another study showed that the N protein inhibits IFN-β production by interacting with IRF3 [36]; however, up to now, studies on PPRV were mostly performed in the human cell line 293T, while humans are no natural PPRV host. 

We hypothesized that PPRV may also inhibit other immune signaling pathways to effectively evade innate immune responses. Interference with the RIG-I-like receptor (RLR) mediated pathways, for instance, might lead to a reduction in the production of ISGs. The activity of this pathway during a PPRV infection was studied by analysing the MAVS adaptor protein in caprine cells. MAVS is crucial in the RIG-I/MDA-5 sensing of various RNA viruses and has been characterized in several species as a key factor in RLR pathways. In our study, we successfully cloned the MAVS homolog from caprine endometrial epithelial cells (EECs), a host cell line for PPRV. By sequence comparison, we found that caMAVS shares conserved structural domains (CARD-PRR-NC-TM) with MAVS of other mammalian species and that caMAVS shares the highest identity with sheep MAVS (98.1%). Further analysis using transiently expressed MAVS mutants mapped individual domains of caMAVS responsible for its distribution in Vero-SLAM cells, which have been described as a useful cell model to study PPRV infection [22]. The full-length caMAVS protein was localized in mitochondria, while caMAVS lacking the TM domain lost the mitochondrial localization and was found in both cytoplasm and nucleus. Further, by using a luciferase-based assay, caMAVS lacking the deleted CARD domain did not induce IFN-β reporter activity and did not activate downstream ISGs. Interestingly, caMAVS carrying a deletion of the NC domain, which has not been studied by others before, also lost mitochondrial localization, but could still induce IFN- β-Luc, NF-kB-Luc, and IRF-3-Luc, albeit less efficiently than the full-length caMAVS. A potential limitation of the luciferase-based assay is the use of HEK 293T cells instead of a natural host cell line (e.g., EECs), but the very low transfection efficiency in such cells precluded us from generating meaningful results. Given that PPRV can replicate in primate cells, the transfection efficiency or reporter plasmids in HEK293T cells is much higher, and the fact that caMAVS overexpression induces IFN-β transcription, we considered this a suitable alternative cell model. Because the induction of downstream signalling by overexpressed caMAVS is independent from interaction with RIG-I, this may explain why deletion of the NC domain abrogates interaction with RIG-I (Figure 6B) but can still induce downstream signalling (Figure 4 and Figure 5). Similarly, in a mouse model of hantaan orthohantavirus (HTNV, genus *Orthohantavirus*, family *Hantaviridae*), it has been observed that IFN production is MAVS independent [37].

By activation of innate immunity, host cells aim to restrict the spread of invading viruses and other pathogens. Once the infection is cleared, cells have their own system to downregulate such responses to return to a normal physiological state. The cellular proteins PCBP2 [38] and PCBP1 [39] can mediate the degradation of the adaptor protein MAVS. NLRX1 (a nucleotide binding oligomerization domain (NOD)-like receptor X1) recruits PCBP2 to induce MAVS degradation through the proteasomal pathway [40]. During HCV infection, the cellular Golgi protein 73 (GP73) acts as a negative regulator of innate immunity by interacting with MAVS/TRAF6 to promote degradation of this complex [41]. Alternatively, viruses may also encode proteins to specifically downregulate MAVS. For example, MAVS is degraded by the Rotavirus (RV) RNA methyl- and guanylyl-transferase (VP3) in a host-range-restricted manner [42]. Likewise, MAVS is cleaved followed by infection with the Avian infectious bronchitis virus [43]. In our study, we observed that PPRV infection induced MAVS degradation in host cells. Moreover, by testing a series of inhibitors, we found that PPRV-induced caMAVS degradation in EECs was medicated by the proteasome. When EECs were infected with a high MOI, the degradation of caMAVS was significant within 36 h post-infection. At the same time, the expression level of a smaller (45–50 kDa) polypeptide that also reacted with the caMAVS antibodies increased from 36 h post infection (Figure 7A), suggesting that caMAVS variants might exist, that may possess diverse biological functions in innate immune regulation. Surprisingly, we also found that the truncated variant is also significantly visible at the 48 h point without virus infection; however, it also decreased later at the 60 h point, which remains unclear. Truncated variants of MAVS (mini MAVS), which is expressed from a bicistronic mRNA of MAVS have been demonstrated to possess the ability to antagonize the signalling function of MAVS and thereby downregulate type I IFN expression by regulation of cell death as described previously [26].

Finally, we showed that overexpression of the PPRV V protein, in absence of a virus infection, also led to the downregulation of caMAVS. Although we did observe co-localization of PPRV-V with caMAVS in transfected cells, further studies will be required to fully understand the degradative interplay between PPRV-V and caMAVS. Interestingly, PPRV-C protein has recently been demonstrated to inhibit IFN-β induction by interacting with RIG-I and MAVS [44], which might indicate that both PPRV-V and PPRV-C are critical for counteracting the innate immune response. Since the results described in this study are based on the PPRV vaccine strain from lineage II, it would be interesting to follow up on this research to investigate commonalities and potential differences between the four circulating lineages [45] in their ability to counteract caMAVS.

In summary, our study characterized a MAVS homolog from immortalized goat cells (EECs) and investigated the functions of caMAVS during type I IFN signalling. We identified caMAVS as a RIG-I interacting type I interferon inducer and showed that caMAVS degraded during PPRV infection, possibly due to an interaction of PPRV-V and caMAVS.

## Data Availability

Data is contained within the article or supplementary material.

## Supplementary Materials

The following are available online at https://www.mdpi.com/1999-4915/13/3/409/s1, Table S1: Primers designed and used in this study; Table S2: The sequence of MAVS selected and included for polygenetic tree construction.
