# Supplementary material for: Caprine MAVS Is a RIG-I Interacting Type I Interferon Inducer Downregulated by Peste des Petits Ruminants Virus Infection
Source: Viruses. 2021 Mar 5;13(3):409. doi: 10.3390/v13030409 (PMC7998690; doi:10.3390/v13030409)
Supplement: Supplementary file 1 [file viruses-13-00409-s001.zip › supplementary/Tabel.s2.docx]

**Table.s2. The sequence of MAVS selected and included for polygenetic tree construction**

| **Species** | **GenBank accession number** |
| --- | --- |
| Ovis aries | XM_015099722.2 |
| Tupaia belangeri | KM005100 |
| Oreochromis niloticus | MG680272 |
| Equus caballus | XM_023626166 |
| Gallus gallus | NM_001012893 |
| Danio rerio | NM_001080584 |
| Rattus norvegicus | BC081869 |
| Callithrix jacchus | KC415025 |
| Papio anubis | NM_001279529 |
| Pan troglodytes | KC415006 |
| Pongo pygmaeus pygmaeus | KC415011 |
| Nomascus leucogenys | KC415010 |
| Gorilla gorilla | KC415007 |
| Mus musculus | DQ174271 |
| chimpanzee | KC415006 |
| Sus scrofa | NM_001097429 |
| Bos taurus | NM_001046620 |
| Felis catus | KT375569 |
| Canis lupus familiaris | NM_001122609 |
