# Supplementary material for: Caprine MAVS Is a RIG-I Interacting Type I Interferon Inducer Downregulated by Peste des Petits Ruminants Virus Infection
Source: Viruses. 2021 Mar 5;13(3):409. doi: 10.3390/v13030409 (PMC7998690; doi:10.3390/v13030409)
Supplement: Supplementary file 1 [file viruses-13-00409-s001.zip › supplementary/Table.s1.docx]

**Table.s1. Primers designed and used in this study**

| Primer name | Primer sequence (5’-3’) |
| --- | --- |
| caMAVS-F | GAGAATTCATGACGTTTGCCGAGGACAGA |
| caMAVS-R | TCGATATCTCACTGGGGTAGGCGCCGCCG |
| caRIG-I-F | AGCAGAGTCGCCGG CATGACGGCC |
| caRIG-I-R | AATTTCTTCATTCAAGGCCATTCATTGCC |
| Caprine-qIFITM3-F | GGGCTATGAGGTGCTCAAGG |
| Caprine-qIFITM3-R | TGAACAGGGACCACACGATG |
| Caprine-qMX1-F | ACTCCCGACTGTTTACCAAAG |
| Caprine- qMX1-R | ACAAACCCTGGCAACTCTC |
| Caprine-qRSAD2-F | GCCCGAGTCTAACCAGAAG |
| Caprine-qRSAD2-R | CTACACCCACGTCCAAGATG |
| Caprine-qOASL-F | GCTGACCCCACCTACAATG |
| Caprine-qOASL-R | AGGACTCTTTCAGGCAATGG |
| Caprine-qGAPDH-F | GATGGTGAAGGTCGGAGTGAAC |
| Caprine-qGAPDH-R | GTCATTGATGGCGACGATGT |
| caMAVS-  △TM-inF | ATGGAGGCCCGAATTCGGATGACGTTTGCCGAGGACAGA |
| caMAVS-  △TM-inR | GTACCTCGAGAGATCTTCACAAGGTCCTGACCACAGGCAA |
| caMAVS-  △CARD-inF | ATGGAGGCCCGAATTCGGATGATCTGTGAGCATACTGGT |
| caMAVS-dTM-inR | GTACCTCGAGAGATCTTCACTGGGGTAGGCGCCGCCGGTA |
| caMAVS-  △PRR-F | \| ATCAGGGCACTGAGGCTGGGCAGTACCCACACAGCAGGCGTG \| \| --- \| \|  \| |
| caMAVS-  △PRR-R | CCTCAGTGCCCTGATGAAGGT |
| caMAVS-△NC-F | CAGGAGCAAGACACAGAATTGCCCTGGGCTCCGTGGCTC |
| caMAVS-△NC-R | TTCTGTGTCTTGCTCCTGATG |
